# Comparison of two doses and two routes of administration of misoprostol after pre-treatment with mifepristone for early pregnancy termination

**DOI:** 10.1186/1742-4755-5-2

**Published:** 2008-06-23

**Authors:** Helena von Hertzen, Gilda Piaggio, Lena Marions

**Affiliations:** 1 UNDP/UNFPA/WHO/World Bank Special Programme of Research, Development and Research Training in Human Reproduction, Department of Reproductive Health and Research, World Health Organization, 20, Avenue Appia, 1211 Geneva 27, Switzerland; 2 Karolinska Hospital, Division of Obstetrics and Gynecology, Department of Woman and Child Health, Stockholm S171-76, Sweden

## Abstract

**Background:**

It is not known whether a 400 μg dose of misoprostol has a similar efficacy as an 800 μg dose when administered sublingually or vaginally 24 hours after 200 mg mifepristone.

**Methods:**

It is proposed to undertake a placebo-controlled, randomized, non-inferiority trial (3% margin of equivalence) of the two misoprostol doses when administered sublingually or vaginally using factorial design. A total of 3008 pregnant women (< 63 days of gestational age) who request legal termination of pregnancy will be recruited for the trial at 16 clinics in ten countries providing abortion services. Eligible women willing to join the study will be allocated randomly to one of the four treatment groups within each centre. Women in all treatment groups will first receive 200 mg mifepristone, followed 24 hours later by either 400 μg or 800 μg misoprostol, administered either sublingually or vaginally. The dose and route of administration of misoprostol will be blinded to women, each woman receiving four tablets vaginally and four tablets sublingually, two or four of which are 200 μg tablets of misoprostol and the rest are placebo tablets.

The four treatment regimens will be compared in terms of: (i) their efficacy to induce complete abortion; (ii) induction-to-abortion interval when possible; (iii) the frequency of side effects; and (iv) women's perceptions. The initial judgment of the outcome of treatment is made at the follow-up visit on day 15 of the study and the final assessment four weeks later. It is estimated that the clinical phase will require 12–14 months for data collection.

To compare the two routes and two doses, relative risks (RR) of failure to achieve a complete abortion and failure to terminate pregnancy and the two-sided 95% CIs will be calculated by standard methods, as well as risk differences and two-sided 95% CIs. The latter will be used to test the non-inferiority hypotheses (at 2.5% level of significance) for achieving complete abortion. The factorial structure will be taken into account in the analysis after testing the interaction.

**Trial registration:**

ISRCTN87811512

## Background

The approved regimen for medical abortion in most countries is 600 mg mifepristone followed 36–48 hours later by 400 μg misoprostol orally up to 49 days of gestation. Between 50 and 63 days, 1 mg gemeprost or 800 μg mg misoprostol administered vaginally is used instead. The data of several multicentre trials conducted over the past ten years demonstrate that for the termination of early pregnancy a single dose of 200 mg of mifepristone is as effective as 600 mg [1-3]. Thus, in the guidelines on safe abortion (Safe Abortion: Technical and Policy Guidance for Health Systems WHO 2003) the recommended regimen is 200 mg mifepristone followed by misoprostol, 400 μg orally (≤ 49 days) or 800 μg vaginally (≤ 63 days).

When misoprostol is administered orally, the dose of 400 μg is used, while 800 μg is administered vaginally. There is no rationale, however, for the use of a higher vaginal dose, because according to pharmacokinetics data and results from studies on uterine contractility, vaginal route should be more effective: compared to oral administration, plasma concentration after vaginal administration stays high for longer [4,5] and the effect on uterine contractility follows the same pattern [6]. There are no studies comparing vaginal doses of 400 μg and 800 μg of misoprostol. Our hypothesis is that vaginal 400 μg will be sufficient and that 800 μg dose is not needed. In fact, if the lower dose has a similar efficacy, it would be unethical to continue using a dose that is unnecessarily high because it is likely to cause more side effects and increase the costs of the treatment.

It has recently been demonstrated that sublingual administration of misoprostol might be an alternative to vaginal treatment. Pharmacokinetic studies have shown that the plasma levels of misoprostol were higher and the area under the curve of misoprostol concentration versus time up to 360 minutes was significantly greater than those following oral and vaginal administration [5]. After sublingual administration the increase in uterine tonus is more rapid and more pronounced than after vaginal administration, but the contractions start to decrease after about 3 hours compared to 4–5 hours after vaginal dose [7,8]. In a pilot study using misoprostol alone [9], a dose of 600 μg was administered sublingually every 3 hours for a maximum of 5 doses to women requesting medical abortion at up to 12 weeks gestation. The overall complete abortion rate was 86%. Diarrhoea, fever and chills were the most common side-effects. Almost all women who had a complete abortion would have chosen this method again. The sublingual route was regarded as convenient and gave more privacy during the abortion process.

In a recent study undertaken in collaboration with our Programme [10], 800 μg misoprostol was administered either vaginally or sublingually after pretreatment with mifepristone for termination of pregnancy. The study included 224 women up to 63 days of gestation. Complete abortion rates were 93.8% and 98.2% after vaginal and sublingual administration, respectively, indicating that the sublingual route could be superior to the vaginal. However, the incidence of side-effects was higher after sublingual administration. Another study also suggested a very high efficacy after sublingual administration of 600 μg of misoprotol among 96 women treated, as 93 of them (98.9%) had a complete abortion [11].

No previous studies have tested 400 μg dose of misoprostol, either sublingual or vaginal, in the first trimester. It does not seems logical to administer a dose for vaginal use that is twice as high as the dose recommended for oral use when scientific data shows the vaginal route to be more efficient. It seems also relevant to test the possibility that sublingual administration might be at least as effective as vaginal administration since a non-vaginal route of administration is preferred by many women [9,12].

The results of our recent study [13] demonstrate an equivalent efficacy for both 24 h and 48 h intervals when 800 μg misoprostol is administered vaginally after mifepristone. As 24 h interval is more practical it is likely to become the standard in mifepristone-misoprostol regimens. This is why we chose the 24 h interval for the present trial.

The ultimate goal of our research is to improve the methods for termination of early pregnancy. The main objective of the present randomized trial is to compare four misoprostol regimens when administered either sublingually or vaginally 24 hours after 200 mg of mifepristone, among women with a gestational age of up to 63 days. The four regimens will be compared in respect of the following main outcomes: (i) their efficacy to induce complete abortion; (ii) induction-to-abortion interval, when possible; (iii) the occurrence of side-effects; and (iv) women's perceptions.

Although there are no comparative studies the efficacy might be somewhat lower if the dose of misoprostol is reduced. If, however, the side-effects will decrease and the regimen is more acceptable to women, it would be justified to use a lower dose if non-inferiority is demonstrated within a margin of 3%. Our hypothesis is that the efficacy of 400 μg misoprostol, whether given sublingually or vaginally after mifepristone pretreatment, is not inferior to that of 800 μg misoprostol within a margin of 3%.

## Methods/design

The design of the study will be placebo-controlled, single-blind, randomized, stratified by centre and by the length of gestation: <49 days; 50–56 days; 57–63 days. A total of 3008 women with pregnancies of < 63 days from last menses (verified by ultrasound) will be recruited for the study in participating centres (188 subjects per centre; 62–64 per each strata of gestation) from among women requesting legal termination of pregnancy and who prefer medical abortion. All subjects will get 200 mg mifepristone orally, and 24 (+/- 2) hours later they will receive either: 4 placebo tablets vaginally and 2 tablets of misoprostol and 2 placebo tablets sublingually; or 4 placebo tablets vaginally and 4 tablets of 200 μg misoprostol sublingually; or 2 tablets of 200 μg misoprostol plus 2 placebo tablets vaginally and 4 placebo tablets sublingually; or 4 tablets of 200 μg misoprostol vaginally and 4 placebo tablets sublingually.

The dose and route of administration of misoprostol will be blinded to the women. Side-effects will be recorded at hourly intervals up to 3 hours after misoprostol administration. The preliminary outcome will be assessed before the woman leaves the clinic. The final outcome regarding the efficacy, side-effects since treatment and acceptability will be assessed at the follow-up visits on Day 15 and Day 42 of the study unless women are still bleeding and require a further follow-up.

This multinational trial will be carried out in 16 departments of obstetrics and gynaecology of teaching hospitals. All departments provide routine abortion services and also carry out clinical research. The trial will be conducted according to ICH-GCP guidelines and the investigators are trained to carry out research accordingly.

A total of 3008 women (188 women at each of the 16 participating centres) will be recruited from among women requesting legal termination of pregnancy during 12 months. Women admitted to the study will fulfil all of the following criteria: good general health; older than the age of legal consent; requesting abortion and eligible for legal termination of pregnancy; on Day 1 of the study (day of mifepristone administration) the duration of pregnancy not more than 63 days, verified by ultrasound; the pregnancy is single and intrauterine (single sac); if treatment with misoprostol should fail agrees to surgical termination of pregnancy; willing and able to participate after the study has been explained; and haemoglobin higher than 90 g/l.

Any indication of serious past or present ill health will be considered a contraindication for recruitment to the study. In particular, women should not be recruited if any of the following conditions is present: allergy towards mifepristone or misoprostol; a history or evidence of disorders that represent a contraindication to the use of mifepristone (chronic adrenal failure, severe asthma uncontrolled by corticosteroid therapy, inherited porphyria) or prostaglandins (mitral stenosis, sickle cell anaemia, diastolic pressure over 90 mm Hg, systolic blood pressure lower than 90 mm Hg measured with a traditional instrument); a history or evidence of thrombo-embolism, severe or recurrent liver disease; has a medical condition or disease that requires special treatment, care or precaution (e.g. corticosteroid or anticoagulant therapy) in conjunction with abortion; uterine fibroids are relative contraindication (women with fibroids that are likely to affect bleeding or contractility should be excluded); the presence of an IUD in utero; breastfeeding; previous surgery of uterus/uterine cervix is a relative contraindication. However, previous low-segment caesarean section does not need to be a contra-indication; suspicion of any pathology of pregnancy (e.g. molar, non-viable pregnancy, threatened abortion); in case difficulties are anticipated in the follow-up of the woman (e.g. lives too far). Women older than 35 years can be recruited for the present trial provided they do not smoke, their diastolic blood pressure is < 90 mmHg and have no known risk factors for cardiovascular disease.

The main efficacy analysis will include all women randomized for whom outcome is known. Criteria for exclusion from per-protocol analysis are the following: essential data are missing from the participant's records making it impossible to judge treatment outcome (same as for main analysis); any violation of the study protocol, including violation of eligibility criteria; and treatment non-compliance.

At each of the participating centres, eligible women will be allocated randomly using blocks of variable size to the four treatment groups using computer-generated random tables. These tables will be produced by the Programme's Statistics and Informatics Services Team. Each participating woman will have two medicine packs: one for Day 1 of the study containing one tablet of 200 mg of mifepristone; and the other for Day 2 of the study (24 hours later) containing 4 tablets to be taken vaginally (either 4 placebo tablets in groups A and B, or 2 misoprostol and 2 placebo tablets in group C, or 4 misoprostol tablets in group D); and 4 tablets to be taken sublingually (either 2 tablets of misoprostol and two placebo tablets in group A; or 4 misoprostol tablets in group B; or 4 placebos in groups C and D). The misoprostol tablets and placebo tablets will be packed in blisters. On the blisters it is indicated which tablets are taken vaginally and which tablets are taken sublingually. All subjects will have the same number of tablets administered vaginally and sublingually. We will use mifepristone (Exelgyn) and misoprostol (Pfizer) tablets which are available in many European countries.

### Study procedures

Before admission to the study potential subjects will undergo the following: ultrasound examination to verify the length of the pregnancy and check that the pregnancy is intrauterine. If the pregnancy is more advanced, if there is a suspicion about an abnormal pregnancy (e.g. extrauterine, molar pregnancy, etc.) the woman will not be admitted to the study; haemoglobin measurement; the blood group and Rh typing according to the routine of the centre; full medical, obstetrical and gynaecological history; full medical and gynaecological examination including pelvic examination, height, weight and blood pressure; and bacteriological examination according to the routine of the centre.

Women who fulfil the criteria for admission and who are willing and able to participate in the trial and have given their informed consent will be included in the study. Women admitted to the study will be given a diary card to record days and amount of vaginal bleeding and side-effects. The card will also give the dates of the follow-up visits. The day of start of the treatment will constitute the day of formal admission to the study (i.e. Day 1 of the study) when the length of pregnancy should not be more than 63 days. The tablet of mifepristone must be swallowed in the presence of a member of the study team who will record on the data form the date and time when the tablet was taken.

All centres will have subject numbers from 1–192. As we plan to take randomly 4 packs of tablets per centre for quality control, each centre will recruit 188 women for the study. To ensure an equal representation of women with longer duration of pregnancy (beyond 49 days) at each centre, subject numbers will be divided between each category of gestational length so that the numbers 1–64 are used for women with the duration of pregnancy up to 49 days, 65–128 for 50–56 days and 129–192 for pregnancies of 57–63 days.

After the admission visit on Day 1 all subjects will attend the clinic on three occasions during the course of the study.

*Day 2 visit: *during this visit which 4 tablets of misoprostol are administered vaginally and 4 tablets sublingually (right after the vaginal dose the first two sublingual tablets, and when they have melted/or after 20 minutes, the additional two tablets sublingually) followed by a 3-hour clinical observation period (hourly recordings of blood pressure, pulse rate, possible side-effects and of any medications given). Both the times of tablet administration and of expulsion of products of conception, if it occurs, will be recorded on the data forms. There will be a vaginal examination at the end of the 3-hour observation period.

*Day 15 visit *is the first follow-up visit. During this visit a medical interview will take place, the diary card is reviewed and a physical and gynecological examination will take place. A blood sample is taken to measure haemoglobin and ultrasound examination is done if judged necessary from clinical findings.

*Day 43 visit *is the second follow-up visit during which the same procedures are carried out as for Day 15, but Hb measurement is not mandatory at this visit. If the woman has not had a spontaneous menstruation by the time of the second follow-up visit, she should have a further follow-up appointment at a date to be decided by the investigator, or she can provide the information by telephone if that is more convenient.

### Assessment of the outcome of treatment

The primary outcome is the efficacy of the treatment in achieving complete abortion and in terminating pregnancy. In addition, the four regimens will be compared in respect of the following outcomes: induction-to-abortion interval, when possible; the occurrence of side-effects; and women's perceptions about the treatments.

#### Efficacy

The efficacy outcome of the treatment will be classified on the basis of the subject's history and the clinical findings at pelvic US examination, as 1) complete abortion – uterus empty; 2) incomplete abortion – remnants of tissue in uterus; 3) missed abortion – intrauterine sac without heart beats; 4) failed termination of pregnancy – live pregnancy, heart beats seen in US; 5) lost to follow, outcome unknown; and 6) Other, including vacuum aspiration on woman's request or when indicated medically before outcome can be assessed.

Even if the woman aborts already after mifepristone, before administration of misoprostol, she will be given misoprostol. The initial judgment about the outcome of therapy is made at the first follow-up visit (Day 15 of the study). If the clinical findings at pelvic examination are suggestive of a continuing pregnancy, an ultrasound examination will be performed. In subjects with live pregnancy (i.e. fetal heart activity present) vacuum aspiration will be performed. In case of missed abortion the investigator may perform vacuum aspiration or continue to follow-up according to the clinical situation.

If the clinical findings at the Day 15 visit are compatible with incomplete abortion, no further action will be taken, unless judged necessary by the investigator, e.g. because of heavy vaginal bleeding or signs of pelvic infection. The final judgment on the outcome of therapy is made at the second follow-up visit on Day 43 of the study. If no emergency or elective curettage was necessary during the period up to the first menstruation, the outcome of treatment is classified as "complete abortion". The remaining cases in which curettage was done will be classified in the first analysis as "incomplete abortion". If the women discontinues the treatment and has vacuum aspiration before the outcome is known, the outcome will be regarded as "undetermined".

#### Induction-to-abortion interval

Induction- to-abortion interval is the time in hours from the start of treatment (mifepristone administration on Day 1) until the expulsion of the products of conception. According to our previous experience up to 70% of the expulsions take place in the clinic during the three-hour observation period after misoprostol administration. The time of expulsion will be recorded on the data collection forms. If expulsion does not occur at the clinic, women are asked to record the time in the daily record charts. It is understood that in very early pregnancies it may not always be possible to identify the time of expulsion.

#### Incidence of side-effects

Signs and symptoms will be recorded at admission, and women will keep daily records of any complaints and medication taken, for the duration of the whole trial and report them at each visit. Data on signs and symptoms will be collected prior to misoprostol administration and immediately after administration as well as at one-hour intervals up to three hours. In addition, the intensity of side effects is recorded indicating whether side-effects were mild, moderate or severe. However, the pain is recorded using the scale from 0–10, in which 0 means no pain and 10 is the worst possible pain the subject can imagine.

We classify the signs and symptoms that women may have during the trial as follows: Pregnancy-related symptoms: such as nausea, vomiting, breast-tenderness, fatigue, dizziness, headache and fainting; Drug-related side-effects, such as diarrhoea, fever and rash; and side-effects related to the abortion process, such as lower abdominal pain and bleeding.

#### Women's perceptions

Women's perceptions on the treatments are assessed at the first follow-up visit using slightly modified versions of the questionnaires from our previous trials.

An individual subject's participation in the study is discontinued if she wants to withdraw her participation or is lost to follow-up. Side-effects such as nausea, vomiting, uterine pain, etc. that may occur in some women during the course of the induced abortion do not constitute a reason for discontinuation and will be treated symptomatically where necessary.

An independent Data Monitoring Committee (DMC) will be appointed to monitor the safety of the study. Its members will discuss the results of interim analyses, will assess whether the magnitude of the difference in the outcomes and side-effects is clinically relevant and/or whether it is associated with the treatment and will give recommendations to the trial Steering Committee regarding stopping the trial prematurely. The ultimate decision about the study discontinuation will be the responsibility of the trial co-ordinator, who without delay will make the information about such a decision available to the participating centres. If any investigator is worried about any symptoms related to the treatment (s)he has to inform the Programme immediately.

The decision whether or not to discontinue a lower dose regimen, because of low efficacy will be based on its comparison with the regimen of 200 mg mifepristone followed by 800 μg misoprostol vaginally 24 hours later. Two interim analyses will be conducted, respectively, when data for 700 subjects are available for analysis in the electronic data file and when data for 1300 subjects are available, or at any point when DMC requests for it. The significance level at the final analysis will be adjusted using Haybittle-Peto rule.

Pre-admission tests and haemoglobin measurement (Days 1 and 15) will be carried out by methods routinely employed at each centre. Ultrasound examinations will be done to verify the length of the pregnancy before admission and, if judged necessary from clinical findings, any time during the study.

### Data management

Participating centres will be requested to record the data on paper data collection forms, enter them and send them electronically on a weekly basis to Geneva, using a Web-based system [14]. Recruitment and data quality will be monitored by the Programme's Statistics and Informatics Services Team using this system.

Data will be analyzed centrally in Geneva. Descriptive statistics will be calculated for all baseline characteristics for all subjects randomized, by treatment group, to describe characteristics of the population and to assess comparability of the groups. The overall summary statistics for women who complete the trial will be compared with those for the subjects lost to follow-up.

All subjects randomized having known outcome will be included in the main efficacy analysis in their randomized groups. If attempts fail to locate and contact a woman lost to follow-up, and her outcome of treatment is not known, then that woman will be excluded from the efficacy analysis. If she has information on side effects, she will be included in the safety analysis. Baseline characteristics of women lost to follow-up will be compared with those completing the study and between groups, to explore the possibility of bias due to these differences.

Crude rates for each outcome will be calculated in each arm and exact confidence intervals based on the binomial distribution calculated as described in Armitage and Berry [15]. If the interaction of misoprostol route by misoprostol dose is significant at 5% level, comparisons will be made between routes for each dose and between doses for each route. If the interaction is not significant at 5%, two comparisons will be made, between routes for the two doses combined, and between doses for the two routes combined. For these comparisons, relative risks (RR) of failure to achieve a complete abortion and failure to terminate pregnancy and the two-sided 95% CIs will be calculated by standard methods, as well as risk differences and two-sided 95% CIs. The latter will be used to test the non-inferiority hypotheses (at 2.5% level of significance) for achieving complete abortion.

Stratified analysis for efficacy will be conducted by gestational age (up to and including 49 days, 50–56 days and 57–63 days). Adjusted analysis by centre and for any prognostic variable showing imbalance at baseline will be performed using Mantel-Haenszel approach and/or logistic regression where appropriate. A per-protocol analysis will be performed excluding women with conditions as described in earlier sections. Efficacy will also be calculated excluding subjects with undetermined outcome. Survival analysis if appropriate will be used to compare the differences in induction-abortion intervals. Chi-square test or Fisher exact test as appropriate, will be used to compare the proportion of women with each side-effect, adjusting for multiple inferences with an appropriate technique.

#### Number of subjects and statistical power

To establish the non-inferiority (one-sided equivalence) of the 400 μg misoprostol (sublingual or vaginal) regimen compared to the 800 μg with respect to efficacy, we require the upper limit of the one-sided 95% confidence interval (or the two-sided 95% confidence interval) for a difference in complete abortion rates (that of 800 μg minus that of 400 μg) to be within a margin of equivalence of 3%. It is estimated that if the abortion rates in the two regimens are both equal to 95%, about 680 subjects will be required in each group to demonstrate non-inferiority with a power of 80%, i.e. a total of about 2720 subjects. To allow for a 5% of women lost to follow-up or with undetermined outcomes, then about 720 women will have to be recruited per group, a total of about 2880. If 15 centres participate in the study, 192 subjects will be needed in each centre. However, to ensure recruitment in one year time, we plan to include one additional centre, 16 centres in all. Since we plan to take four packs per centre for quality control, the actual number of women recruited per centre will be 188. These calculations allow for the presence of an interaction between misoprostol dose and its route of administration.

For the choice of the non-inferiority margin, a clinical criterion was used, not a statistical one. This is based on the fact that there are not placebo-controlled trials available and the efficacy of placebo would be close to zero. We set the margin at 3% based on clinical assessment that this difference is not relevant.

It is estimated that the trial will require 12 to 14 months for data collection at each centre and between 6 and 12 months of centralized data analysis in Geneva.

#### Project management

The trial will be conducted in 16 collaborating centres under the supervision of a principal investigator in each centre. The responsibilities of principal investigators are described in the "Guidelines for Investigators" for this project. Overall co-ordination of the project will be the responsibility of the Manager and statistical staff of the Programme's Group for Research on Post-ovulatory Methods for Fertility Regulation. This responsibility will include, inter alia, the organization and supervision of the trial, communication with the principal investigators, and the analysis and writing-up of the results.

This trial will be funded by the Packard Foundation and the UNDP/UNFPA/WHO/World Bank Special Programme of Research, Development and Research Training in Human Reproduction, Department of Reproductive Health and Research, World Health Organization.

## Authors' contributions

HvH and LM drafted the protocol with statistical input by GP. All authors read and approved the final protocol.
